# Identification of Small Molecule Inhibitors of the Pathogen Box against Vibrio cholerae

**DOI:** 10.1128/Spectrum.00739-21

**Published:** 2021-12-22

**Authors:** Haeun Kim, Brianne J. Burkinshaw, Linh G. Lam, Kevin Manera, Tao G. Dong

**Affiliations:** a Department of Ecosystem and Public Health, Faculty of Veterinary Medicine, University of Calgary, Calgary, Alberta, Canada; b Biochemistry and Molecular Biology, Cumming School of Medicine, Snyder Institute for Chronic Diseases, University of Calgary, Calgary, Alberta, Canada; c Department of Immunology and Microbiology, School of Life Sciences, Southern University of Science and Technology, Shenzhen, Guangdong, China; Forschungszentrum Jüclich GmbH

**Keywords:** antibiotics, Pathogen Box, cholera, AMR, small molecule

## Abstract

Antimicrobial resistance (AMR) has become a serious public and economic threat. The rate of bacteria acquiring AMR surpasses the rate of new antibiotics discovery, projecting more deadly AMR infections in the future. The Pathogen Box is an open-source library of drug-like compounds that can be screened for antibiotic activity. We have screened molecules of the Pathogen Box against Vibrio cholerae, the cholera-causing pathogen, and successfully identified two compounds, MMV687807 and MMV675968, that inhibit growth. RNA-seq analyses of V. cholerae after incubation with each compound revealed that both compounds affect cellular functions on multiple levels including carbon metabolism, iron homeostasis, and biofilm formation. In addition, whole-genome sequencing analysis of spontaneous resistance mutants identified an efflux system that confers resistance to MMV687807. We also identified that the dihydrofolate reductase is the likely target of MMV675968 suggesting it acts as an analog of trimethoprim but with a MIC 14-fold lower than trimethoprim in molar concentration. In summary, these two compounds that effectively inhibit V. cholerae and other bacteria may lead to the development of new antibiotics for better treatment of the cholera disease.

**IMPORTANCE** Cholera is a serious infectious disease in tropical regions causing millions of infections annually. Vibrio cholerae, the causative agent of cholera, has gained multi-antibiotic resistance over the years, posing greater threat to public health and current treatment strategies. Here we report two compounds that effectively target the growth of V. cholerae and have the potential to control cholera infection.

## INTRODUCTION

Most antibiotics that we currently use are molecules naturally produced by microbes to survive in complex microbial communities (1). Therefore, it is only natural for these microbes to be resistant against different antibiotics to outcompete their opponents and neighbors (2). However, antibiotic resistance has become a serious public threat due to an increased rate of microbes gaining resistance against not one, but multiple antibiotics. It was reported that approximately 700,000 people die annually due to complications with antibiotic-resistant infections (3). In the United States alone, two million patients suffer from multi-drug resistant (MDR) infections annually, incurring a cost of $20 billion to the health care system (3). In June 2019, the Public Health Agency of Canada announced that there is a 1-in-16 chance of developing a superbug (a bacterium resistant to most commonly available antibiotics) infection while being hospitalized (4). There is also an increase in the number of Canadians carrying bacteria resistant to carbapenem, one of the last line of defense antibiotics (4).The problem of antibiotic resistance requires immediate attention to prevent further damage to the world’s population and economy (3).

Vibrio cholerae is a rod-shaped, Gram-negative bacterium that causes a serious diarrheal disease, cholera (1, 5–7). After being ingested through contaminated food or water, V. cholerae colonizes the small intestine of the infected host (7). Its secreted cholera toxin causes severe rice-water diarrhea due to the uncontrolled movement of water into the small intestine (7). There have been seven pandemics of cholera with the first pandemic recorded in 1817 and the seventh pandemic still ongoing in the areas of Africa and Asia (8, 9). Pandemic-causing V. cholerae strains all belong to the O1 serogroup, which is further divided into two biotypes: the Classical strains that caused the first six pandemics and the El Tor strains that caused the current pandemic and replaced the Classical strains (6).

Even though cholera can be mitigated through proper hygiene and access to clean water, it remains one of the major health problems in developing countries around tropical regions (10). It has been estimated that 1.3 to 4 million people suffer from cholera annually with tens of thousands of deaths (6, 10). Cholera is mostly treated by administering patients with oral rehydration solution (ORS) (10). However, in severe cases, antibiotics such as tetracyclines and quinolones are administered together to lessen the symptoms especially in children aged 5 and under (7). Although antibiotics are not the first line of treatment for cholera, the use of antibiotics is critical for countries with endemic cholera and ongoing epidemics, especially in the countries with limited access to clean water and ORS supplies (7).

As discussed above, bacteria with acquired antibiotic resistance pose a global threat, and V. cholerae is not an exception. V. cholerae’s resistance to antibiotics was first described in the 1960s, which was due to spontaneous mutations in the drug targets. More recently, MDR V. cholerae is on the rise due to lateral gene transfer, particularly through the movement of integrating conjugative elements, leading to a rise of various strains of MDR V. cholerae among clinical and environmental isolates (1, 11). It is clear that there is an ever-increasing need to identify more efficient antibiotics against this notorious bacterium.

Pathogen Box is a library of 400 drug-like compounds that have been shown to target neglected tropical diseases like kinetoplastid infections as well as malaria and tuberculosis (12). These compounds, with known cytotoxicity and pharmacokinetics, serve as an important resource for drug development against a broad range of pathogens (13, 14). In this study, we have screened the library and identified two small molecules, MMV687807 and MMV675968, that individually target different biological processes to either kill or inhibit V. cholerae growth. We report that both compounds cause significant transcriptomic changes to a clinical isolate of V. cholerae, strain C6706. We also show that a mutation in *vc1408*, a gene encoding for a negative regulator of an efflux pump, confers resistance to MMV687807. Furthermore, we present strong evidence that MMV675968 could be targeting the dihydrofolate reductase of V. cholerae with a MIC 14-fold lower than trimethoprim (an analog of the compound). These findings may not only facilitate effective treatment of cholera disease but also lead to discovery of effective compounds against other MDR-related infections.

## RESULTS

### Discovery of Vibrio cholerae-inhibiting compounds by screening the Pathogen Box.

**FIG 1:**
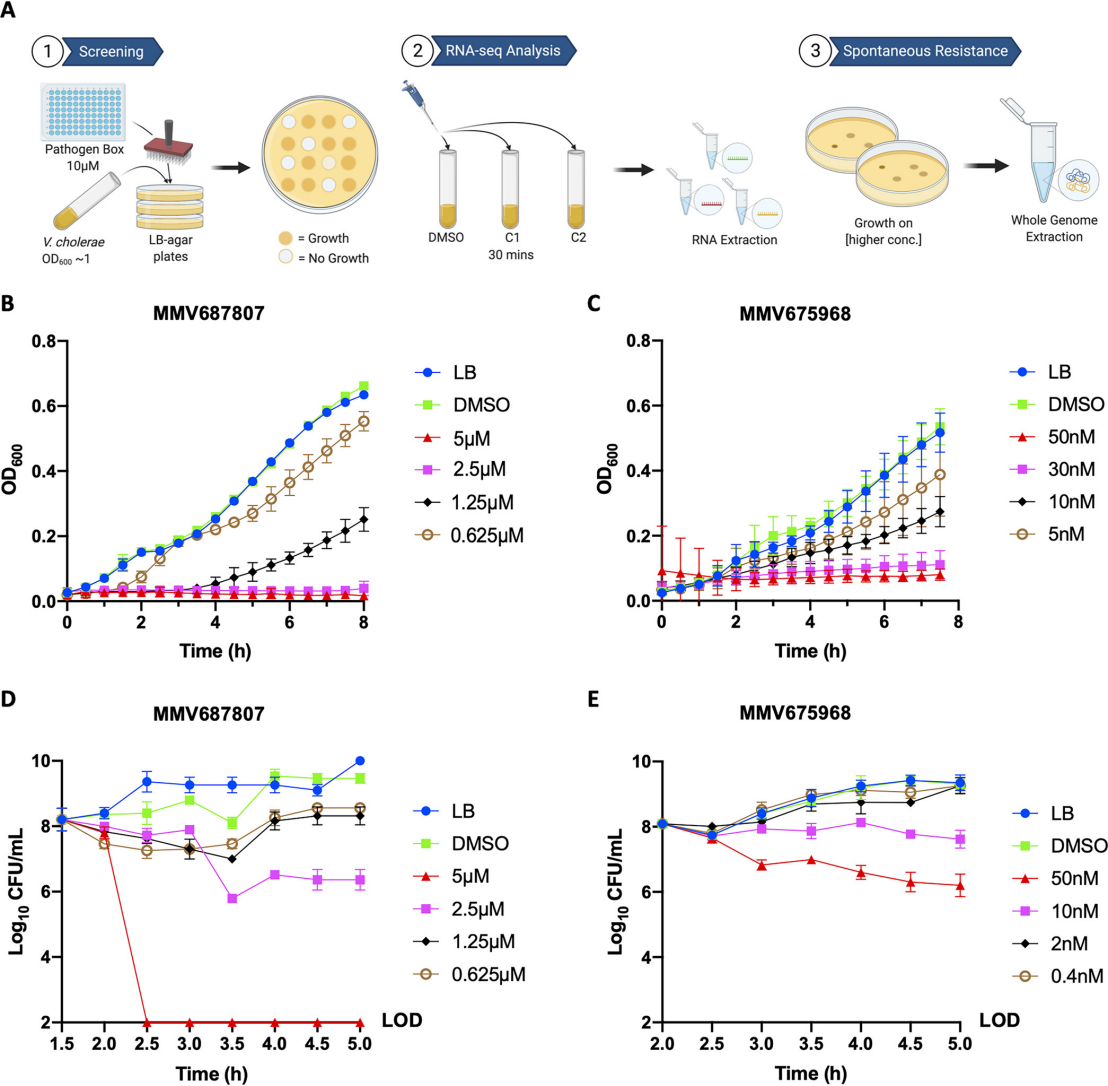
V. cholerae C6706 is either killed or inhibited by MMV687807 and MMV675968, respectively. (A) Schematic of screening Pathogen Box compounds against C6706. C1 indicates MMV687807, and C2 indicates MMV675968. OD_600_ growth curve measurements of C6706 cells inhibited with varying concentrations of (B) MMV687807 or (C) MMV675968. Legend indicates the concentration of compound used. Log_10_ CFU/mL of C6706 after growing with varying concentrations of (D) MMV687807 or (E) MMV675968. Cells were grown to an OD_600_ of 0.4 before the addition of each compound or DMSO. 10-fold serial dilution of cells was plated on LB-agar for counting colonies at each time point. LOD, limit of detection. Error bars indicate mean ± standard deviation of three biological replicates.

V. cholerae El Tor strain C6706, referred as C6706 hereafter, is a seventh-pandemic strain isolated from Peru in 1961 (15, 16). To identify inhibitors of C6706, we screened 400 compounds of the Pathogen Box at 10 μM concentration (Fig. 1A). While most compounds showed no growth inhibition, six compounds exhibited a zone of clearing, indicating inhibition of growth (Table 1). Out of the six compounds identified, MMV687807 and MMV675968 showed the most potent inhibiting activities, suppressing growth by 50% or more after 8 h at concentrations of 1.25 μM and 10 nM, respectively (Fig. 1B and C). We further focused on these two compounds as they showed the greatest inhibition but are also yet to be commercially used.

**TABLE 1:** Pathogen Box compounds that inhibit growth of V. cholerae at 10 μM concentration

Identification	Common name	Formula/structure^*a*^	Commercial use	Pathogen Box target^*a*^	HepG2 IC20 (μM)^*a*^	Mode of action
MMV687807	NA	C15H8NO2ClF6 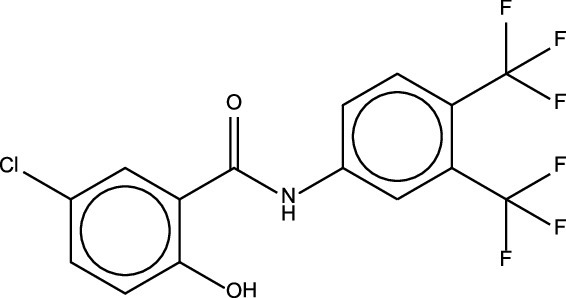	NA	Mycobacterium tuberculosis	0.658	unknown
MMV675968	NA	C17H18N5O2Cl 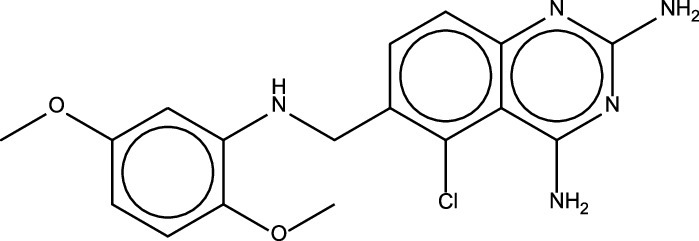	NA	Cryptosporidium parvum	3.44	Inhibition of DHFR
MMV019993	NA	C17H17N6F3 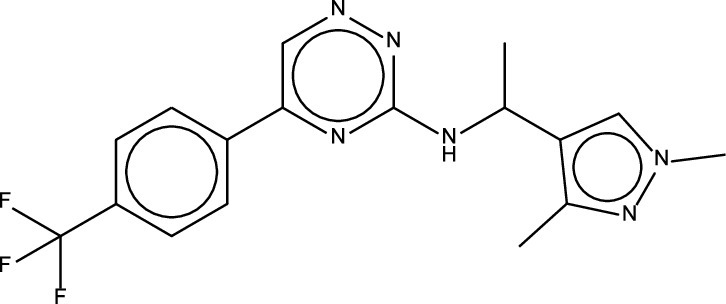	NA	Plasmodium falciparum	39.3	unknown
MMV002817	Iodoquinol	C9H5NOI2 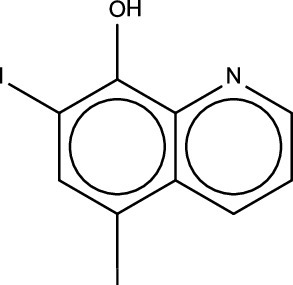	amebicide	Brugia pahangi	2.53	Chelation of ferrous ions
MMV000011	Doxycycline	C22H24N2O8 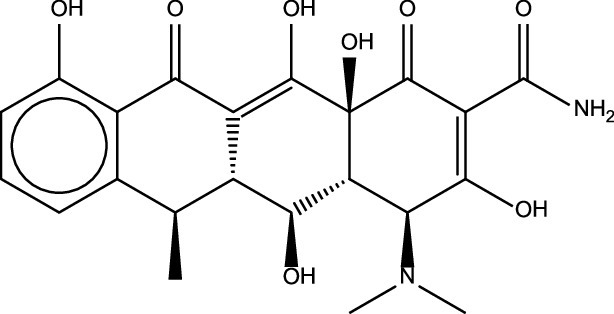	antibiotic	broad range spectrum	18.2	Inhibition of protein synthesis
MMV687798	Levofloxacin	C18H20N3O4F 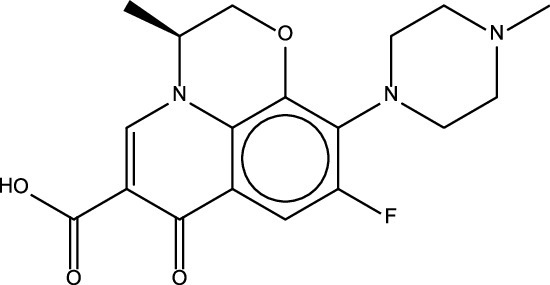	antibiotic	broad range spectrum	>80	Inhibition of DNA gyrase and topoisomerase IV

a Information provided by Medicines for Malaria Venture.

To gain a deeper understanding of growth inhibition after treatment with different concentrations of the compounds, we employed serial dilution plating and enumeration of CFU per mL (CFU/mL) at numerous time points. At 5 μM, MMV687807 showed bactericidal activity since it brought the CFU/mL counts below the limit of detection (Fig. 1D). In contrast, MMV675968 showed a more modest reduction in surviving colonies, suggesting a predominantly bacteriostatic activity at the concentrations tested (Fig. 1E). We further examined the activities of these compounds by treating non-dividing cells with these compounds. Neither compound exhibited antibacterial activities when cells were in stationary phase compared to cells treated during exponential phase (Fig. S1). This suggests that both compounds target a system involved in active growth and cell division.

### Both compounds modulate multiple biological functions.

To gain insight into the molecular mechanisms of inhibition, we performed RNA-seq analyses of C6706 exposed to sub-inhibitory concentrations of each compound, 2.5 μM MMV687807 and 10 nM MMV675968, then grouped differentially expressed genes based on biological functions.

**FIG 2:**
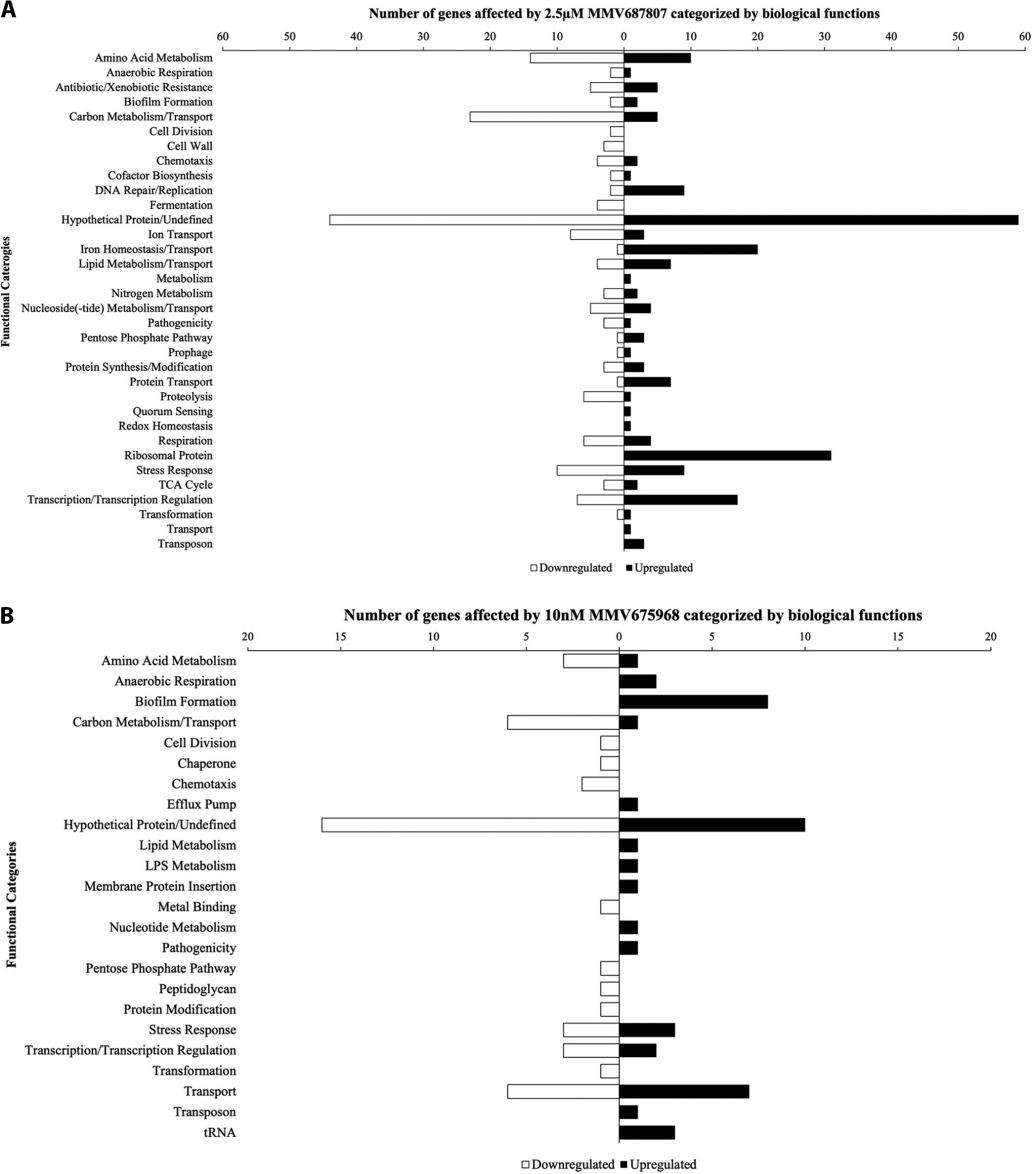
Classification by functional categories of the genes that are significantly upregulated or downregulated when treated with sub-inhibitory concentrations of (A) MMV687807 (*n* = 387) and (B) MMV675968 (*n* = 91). Each set of RNA-seq data was compared to the cells exposed to the DMSO control (0.2%). Differentially expressed genes were identified using DESeq2 and filtered for adjusted *P value* < 0.05. Genes with differential expression values were selected at log_2_ ≥ 2 or log_2_ ≤ −2 for MMV687807 and log_2_ ≥ 1 or log_2_ ≤ −1 for MMV675968. Functional categories of the genes were identified through KEGG pathway mapper and UniProtKB.

When cells were exposed to 2.5 μM MMV687807, 387 genes were identified with adjusted *P value* < 0.05 and differential expression values greater than or equal to 4-fold (log_2_ ≥ 2 or log_2_ ≤ -2). Each of the identified genes was classified into their biological functions with 103 genes falling under hypothetical proteins with unknown functions (Fig. 2A). The most downregulated group was genes involved in carbon metabolism/transport. A few of the most downregulated genes, *vc1820*, *vc1826*, and *vc1821*, are involved in the uptake of fructose. Iron homeostasis/transport showed the most upregulated number of genes along with ribosomal proteins (Fig. 2A). These include *vca0676* and *vc1184*, involved in the formation of iron-sulfur clusters, and *vc0364* and *vc0475*, which have iron storage and siderophore activities. This analysis revealed that MMV687807 has a very broad effect on the cells. It is possible that these transcriptomic changes are an indirect result of the compound targeting an essential system of the cell or due to the result of targeting multiple cellular pathways.

Cells exposed to 10 nM MMV675968 exhibited 91 genes that are differentially expressed with values greater than or equal to 2-fold (log_2_ ≥ 1 or log_2_ ≤ -1) and adjusted *P value* < 0.05. When these genes were classified into biological functions; 26 genes were classified as hypothetical proteins with unidentified functions (Fig. 2B). The category of biofilm formation showed the most upregulated genes. However, when biofilm formation was quantified using crystal violet assay, there was no significant difference between the dimethyl sulfoxide (DMSO) control and different concentrations of MMV675968 (Fig. S2). Additionally, genes involved in exopolysaccharide formation, like *vc0937* and *vc0934*, were also upregulated. Similar to MMV687807, genes involved in transport of carbon or small molecules showed most downregulation (Fig. 2B). We additionally found a few stress response genes that had significant upregulation but with modest and less than 2-fold changes in expression (Table S1). These included genes involved in stringent response (*rpoZ*) and envelope stress response (*rpoE and rseABC*). Specifically, we also discovered that a gene involved in SOS response (*uvrA*) was upregulated as well. This suggests that the target of MMV675968 could be involved in pathways maintaining proper membrane structure and DNA repair.

Next, we compared the RNA-seq analyses between the compounds in order to see if there were genes and/or pathways that are similarly affected by both compounds. Nineteen genes were differentially expressed under both treatment conditions (Table S2). The genes belong to multiple functional categories including stress response, carbon metabolism and transport, and hypothetical proteins. Notably, a number of genes that were downregulated in the presence of MMV687807 showed upregulation in the presence of MMV675968 suggesting that these two compounds have different modes of action when inhibiting the growth of C6706.

### Whole genome sequencing of spontaneous resistance mutants.

To gain further insight into the molecular targets of these compounds, we obtained spontaneous resistance mutants by plating C6706 cells on LB plates containing each compound. Spontaneous resistance mutants were purified on a new LB plate with appropriate concentrations of each compound and then subjected to whole genome sequencing analysis using the Illumina HiSeq4000 platform.

Three spontaneous mutants resistant to MMV687807 at 3 μM or 5 μM were selected. Whole genome sequencing revealed multiple mutations in *vca0792* and *vca0791*, encoding subunits of transposase OrfAB; however, these were found in all sequenced mutants suggesting that they reflect the genome background differences between our C6706 strain and the reference V. cholerae isolate N16961. More promisingly, *vc1408* showed a frameshift mutation in one of the resistant mutants. This gene encodes an HTH transcriptional regulator VceR, a negative regulator of the efflux pump VceCAB (17). On the other hand, two resistant mutants were selected from cells grown at 25 nM MMV675968. Both mutants showed a mutation in *vca0767*, a gene responsible for another HTH transcriptional regulator with an unknown target.

**FIG 3:**
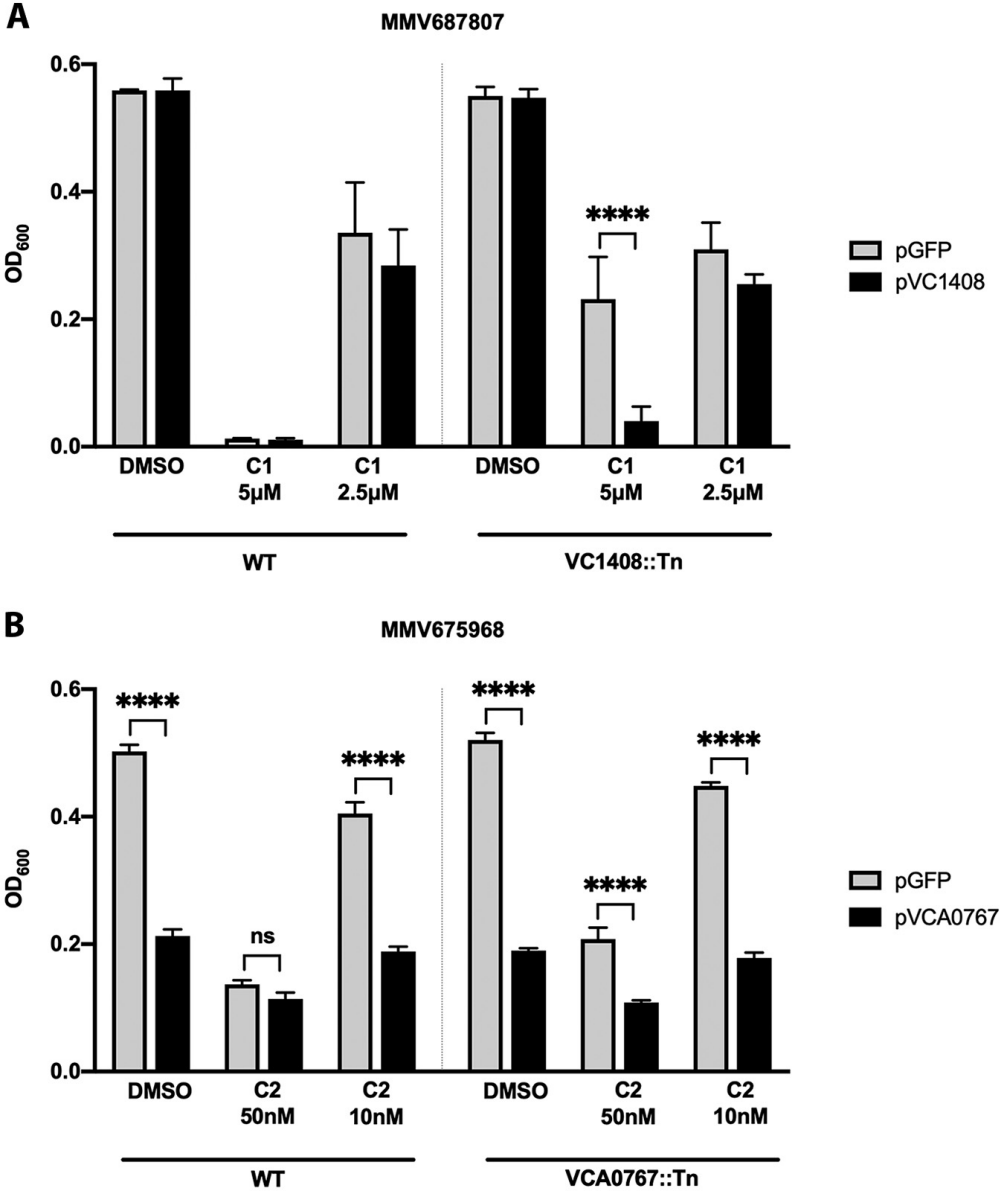
Effects of transposon mutations of genes affected in spontaneous resistance mutants on the growth of C6706 during treatment with each compound. (A) Effect of mutation of VC1408 on the survival of C6706 treated with MMV687807 (C1). (B) Effect of mutation of VCA0767 on the survival of C6706 treated with MMV675968 (C2). C6706 was transformed with an arabinose-inducible plasmid containing each of the indicated genes. Transformants were grown overnight and diluted to OD_600_ ∼0.05 in fresh LB with indicated concentrations of each compound and 0.2% arabinose. Each bar represents OD_600_ after an 8-h growth in a 96-well plate. Comparisons between the strains expressing GFP and gene of interest were done using one-way ANOVA with Sidak’s multiple comparison, ****, *P* < 0.0001, ns, not significant. Error bars indicate mean ± standard deviation of three biological replicates.

To confirm that the mutation in these identified genes leads to resistance against these two compounds, we transformed WT C6706 and transposon mutants of each gene with an arabinose-inducible plasmid containing each of our genes of interest (*vc1408* and *vca0767*). When *vc1408* was induced in the VC1408::Tn mutant, the cells lost resistance to MMV687807 compared with the cells expressing an empty plasmid (Fig. 3A). Although the strains induced with *vca0767* show significantly reduced protection from MMV675968, this is likely due to toxicity of *vca0767* overexpression as both wild type and transposon mutant overexpressing *vca0767* presented significantly reduced growth in the untreated control (Fig. 3B). On the other hand, we can confirm that disruption of *vc1408* instigates resistance. This is likely through de-repression of the VceCAB efflux pump (17, 18).

### MMV675968 targets the dihydrofolate reductase.

**FIG 4:**
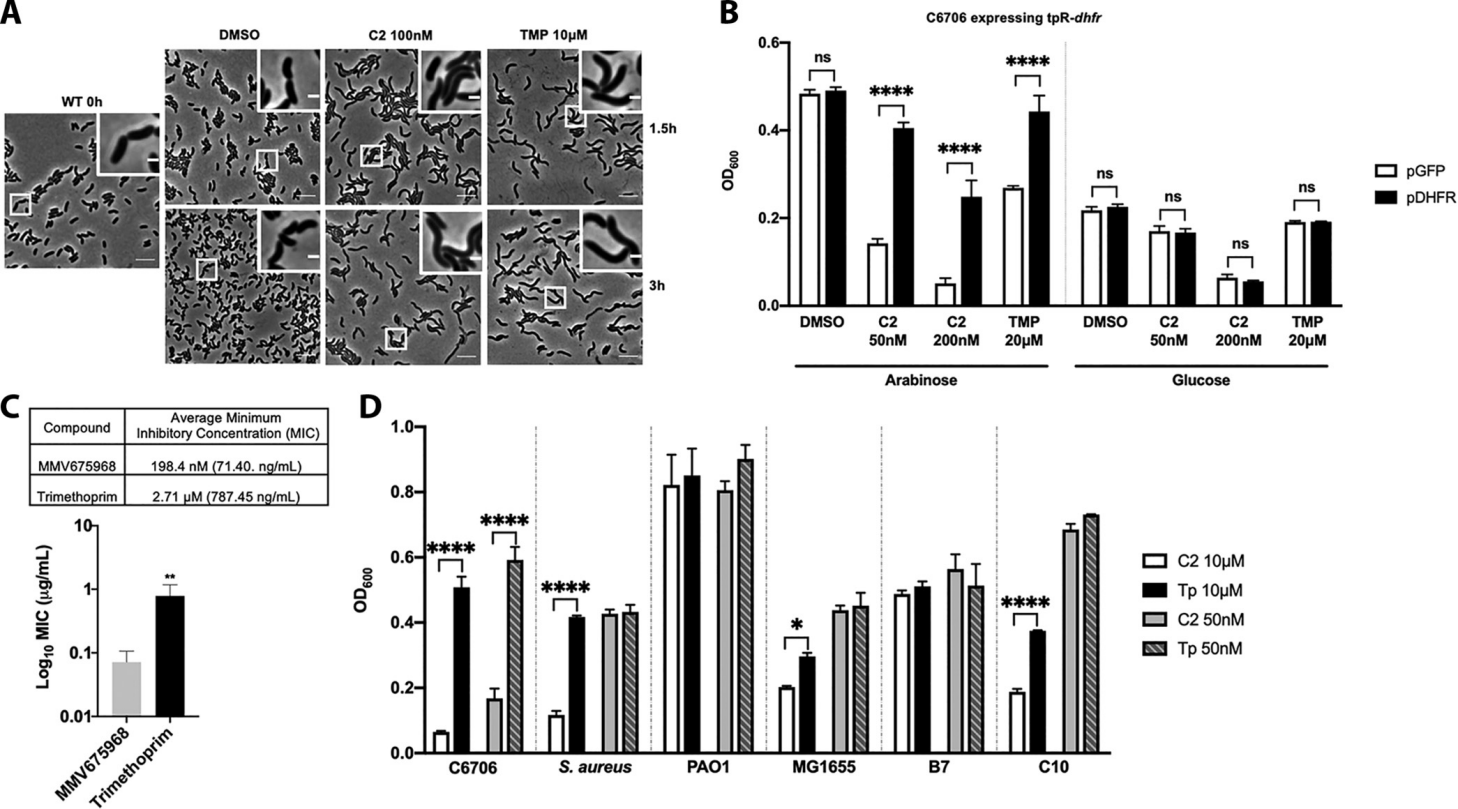
MMV675968 targets the DHFR of C6706 with better efficacy. (A) Phase contrast images of C6706 exposed to DMSO, MMV675968 (C2), or trimethoprim (TMP). Representative image of 40- × 40-μm field of cells with a magnified 5- × 5-μm inset of a selected region shown. Scale bar is 5 μm for the original field of view and 1 μm for the inset. (B) Effect of trimethoprim-resistant *dhfr* (TpR-*dhfr*) on the survival of C6706 when treated with MMV675968 (C2) or trimethoprim (TMP). C6706 was transformed with an arabinose-inducible plasmid containing TpR-*dhfr*. Transformants were grown overnight and diluted to OD_600_ ∼0.05 in fresh LB with indicated concentrations of each compound and 0.2% arabinose for expression or 0.4% glucose for repression. Each bar represents OD_600_ after an 8-h growth in a 96-well plate. One-way ANOVA with Sidak’s multiple comparison was used to compare the growth of cells expressing TpR-DHFR and GFP under each condition, ****,*P* < 0.0001; ns, not significant. Error bars indicate mean ± standard deviation of three biological replicates. (C) MIC comparison between trimethoprim and MMV675968. C6706 was grown in CAMHB with varying concentrations of each compound overnight. MIC was determined by observing for a concentration that did not present growth to the naked eye. To compare the observed MIC between the two compounds, an *unpaired t test* was used, **, *P* < 0.05. Representative data from three biological replicates are shown. (D) Effect of MMV675968 across multiple species. Each species were grown overnight, normalized to OD_600_ ∼2, and diluted to OD_600_ ∼0.05 in fresh LB with indicated concentrations of MMV675968 (C2) or trimethoprim. (Tp). Each bar represents OD_600_ after an 8-h growth in 96-well plate. One-way ANOVA with Sidak’s multiple comparison was used to compare the treatments between MMV675968 and trimethoprim, *, *P* < 0.0332; ****, *P* < 0.0001. Error bars indicate mean ± standard deviation of three biological replicates.

A recent study has shown that MMV675968 targets dihydrofolate reductase (DHFR) in Acinetobacter baumannii (another Gram-negative pathogen with strains resistant to numerous antibiotics) in a manner similar to how trimethoprim targets DHFR (19). We speculated that a similar mechanism could be causing growth inhibition in C6706. We hypothesized that if MMV675968 targets DHFR, it will cause C6706 to elongate similar to trimethoprim causing cell elongation (20, 21). As expected, cells exposed to high concentrations of both MMV675968 and trimethoprim showed obvious cell elongation, indicating disrupted cell division (Fig. 4A).

To further support that MMV675968 could be targeting DHFR, we transformed C6706 with arabinose-inducible plasmids containing either *sfgfp* (empty plasmid) or trimethoprim resistant *dhfr* (TpR-*dhfr*). Expressing TpR-*dhfr* in C6706 was able to rescue the growth of cells treated with high concentrations of both MMV675968 and trimethoprim, while GFP expression had no impact (Fig. 4B). Additionally, when plasmid expression was repressed by the addition of glucose, no growth difference was observed between empty plasmid and TpR-*dhfr* containing strains. We also studied whether MMV675968 is able to inhibit growth of C6706 at a lower concentration than trimethoprim using the broth microdilution method (22). Trimethoprim exhibited growth inhibition at a concentration around 2.71 μM whereas MMV675968 showed inhibition around 198.4 nM, a concentration 14-fold lower than trimethoprim (Fig. 4C).

Finally, we tested whether MMV675968 targets bacteria as broad as trimethoprim. Pseudomonas aeruginosa PAO1 and trimethoprim-resistant MDR Escherichia coli (B7) did not exhibit growth reduction. However, a Gram-positive bacterium, Staphylococcus aureus, treated with 10 μM MMV675968 showed significant growth inhibition compared to trimethoprim-treated cells. We also observed growth inhibition of E. coli MG1655 and non-trimethoprim-resistant MDR E. coli (C10) (Fig. 4D). These results suggest that MMV675968 may be an improved substitute for trimethoprim across multiple species including C6706.

## DISCUSSION

V. cholerae is a pathogen that causes the serious diarrheal disease, cholera. Although cholera can be prevented and treated by maintaining good hygiene and having access to clean water, developing countries still suffer from thousands of deaths annually (10). Antibiotics are often administered together with rehydration solution during cholera treatment to lessen the severity and duration of the symptoms. As V. cholerae has become resistant to many of the commonly used antibiotics like trimethoprim-sulfamethoxazole and chloramphenicol, there is an increasing need for new antibiotics against this pathogen (23). In this study, we have screened the Pathogen Box and identified two compounds, MMV687807 and MMV675968, that either kill or inhibit the growth of V. cholerae El Tor strain C6706 by at least 50% at concentrations as low as 2.5 μM and 10 nM, respectively.

MMV687807 was first identified as a derivative of IMD-0354, a salicylamide that is in clinical trials for atopic dermatitis (eczema) and is also known to suppress the development and metastasis of colon cancer (12, 24). MMV687807 not only shows comparable activities to IMD-0354 but also targets multiple pathogens like Mycobacterium tuberculosis and Candida albicans though its mechanism of action is not fully understood (25, 26).

We show that treatment with MMV687807 causes global changes in the transcriptome, causing downregulation of many genes involved in amino acid metabolism and carbon metabolism, whereas genes involved in iron homeostasis were upregulated (Fig. 2A). A common response of cells exposed to bactericidal antibiotics is a reduced rate of carbon metabolism, which decreases sensitivity to well-known antibiotics like ampicillin (27–29). It has also been shown that disrupting the transcriptional regulator of iron homeostasis increases antibiotic resistance (30). These previous findings could explain the observed changes after the exposure to the compound. These responses allowed the cells to survive during the treatment period. However, this analysis does not give the primary mode of action for the compound because these changes seem to be global across different species exposed to various antibiotics and are secondary to the compound’s mode of action.

We show that a transposon mutation in a repressor gene of the efflux pump VceCAB causes C6706 to become resistant to MMV687807 (Fig. 3A). VceCAB is one of the efflux pumps found in V. cholerae that plays an important role in excreting toxic molecules out of the cell (7). Substrates of VceCAB include multiple antibiotics such as chloramphenicol, a proton motive force uncoupler carbonyl cyanide *m*-chlorophenylhydrazine (CCCP), and salicylate (17, 18). It has been shown that VceR, the product of *vc1408*, normally represses the expression of VceCAB (17, 18) Interestingly, VceR switches between DNA-binding and non-DNA-binding conformations depending on the interaction with its substrates, like CCCP, through competition with dsDNA in a concentration-dependent manner (31). Because disruption of VceR in C6706 led to strong resistance against MMV687807 at varying concentrations, it suggests that de-repressed VceCAB is able to efflux MMV687807.

Although MMV687807 seems to be a promising new option for treating cholera, there are many other aspects, such as cellular toxicity, that need to be considered before it can be further developed into a commercially viable drug. Medicines for Malaria Venture (MMV), the provider of Pathogen Box, provides critical information on drug metabolism and pharmacokinetics of each compound. MMV687807 has been shown to only require 0.658 μM to reach a concentration of 20% inhibition (IC20) in human hepatoma cells (HepG2) (Table 1). This concentration is lower than the effective concentration against V. cholerae. Further studies are required to fully understand the target of this compound to improve its utility.

Similarly, MMV675968 is also able to inhibit multiple pathogens like Cryptosporidium parvum, Plasmodium falciparum, and A. baumannii (19, 32, 33). Multiple studies have shown that MMV675968 acts by targeting the DHFR and could be a potential replacement for trimethoprim (34, 35). We also provide evidence that DHFR may be a target of MMV675968 in V. cholerae.

MMV675968 treatment caused a noticeable downregulation of genes involved in carbon metabolism/transport (Fig. 2B). We speculate that this is similar to how MMV687807 affected the carbon pathways. An interesting observation of this compound’s effect is that there was a significant increase in the expression of the genes involved in biofilm formation. Biofilm formation plays an important role in the pathogenicity and the survival of V. cholerae when living in a free environment where residual concentration of antibiotics can be commonly found (5). The upregulation of genes involved in biofilm formation may protect against an attacking antibacterial molecule by limiting the rate of compound penetration into the cells. In addition, we have also observed upregulation of genes involved in stress-induced proteolysis (36), sigmaE stress response (36, 37), DNA repair (38). These findings suggest that MMV675968 could be directly or indirectly affecting pathways involved in cell growth and division.

Trimethoprim was discovered in the 1960s as a 2,4-diaminopyrimidine that inhibits a broad-spectrum of bacteria and exhibits minimal mammalian toxicity (39). In the 1980s, the structure of trimethoprim binding to its ligand was extensively studied and dihydrofolate reductase (an essential enzyme that reduces dihydrofolate into tetrahydrofolate which is used in nucleic acid synthesis) was identified as its target (40, 41). When its effectiveness was studied against V. cholerae, the MIC against El Tor strains were 1.7 to 2.0 μg/mL (42). Our MIC test of trimethoprim against C6706 showed a similar range with average MIC of 2.71 μM (787.45 ng/mL) (Fig. 4C). However, V. cholerae resistance to trimethoprim has been reported to be through acquiring a resistance gene via mobile genetic materials like integrating conjugative elements (1, 7). Interestingly, MMV675968, an analog of trimethoprim, works much more effectively against C6706 than trimethoprim at a MIC 14 times lower in molarity, 198.4 nM (71.4 ng/mL). Furthermore, MMV675968 exhibits a broad range of inhibiting activities against multiple species including S. aureus and MDR E. coli (Fig. 4D). How exactly MMV675968 exhibits inhibition at lower concentrations than trimethoprim is to be further studied. Unlike MMV687807, MMV675968 has greater potential for further development as a new treatment for cholera. MMV data have shown that HepG2 IC20 of this compound is 3.44 μM (Table 1), much higher than the effective anti-*Vibrio* concentration (50 nM). Further study is needed for testing any synergistic effects it may have when used with sulfamethoxazole, an antibiotic commonly used with trimethoprim.

**FIG 5:**
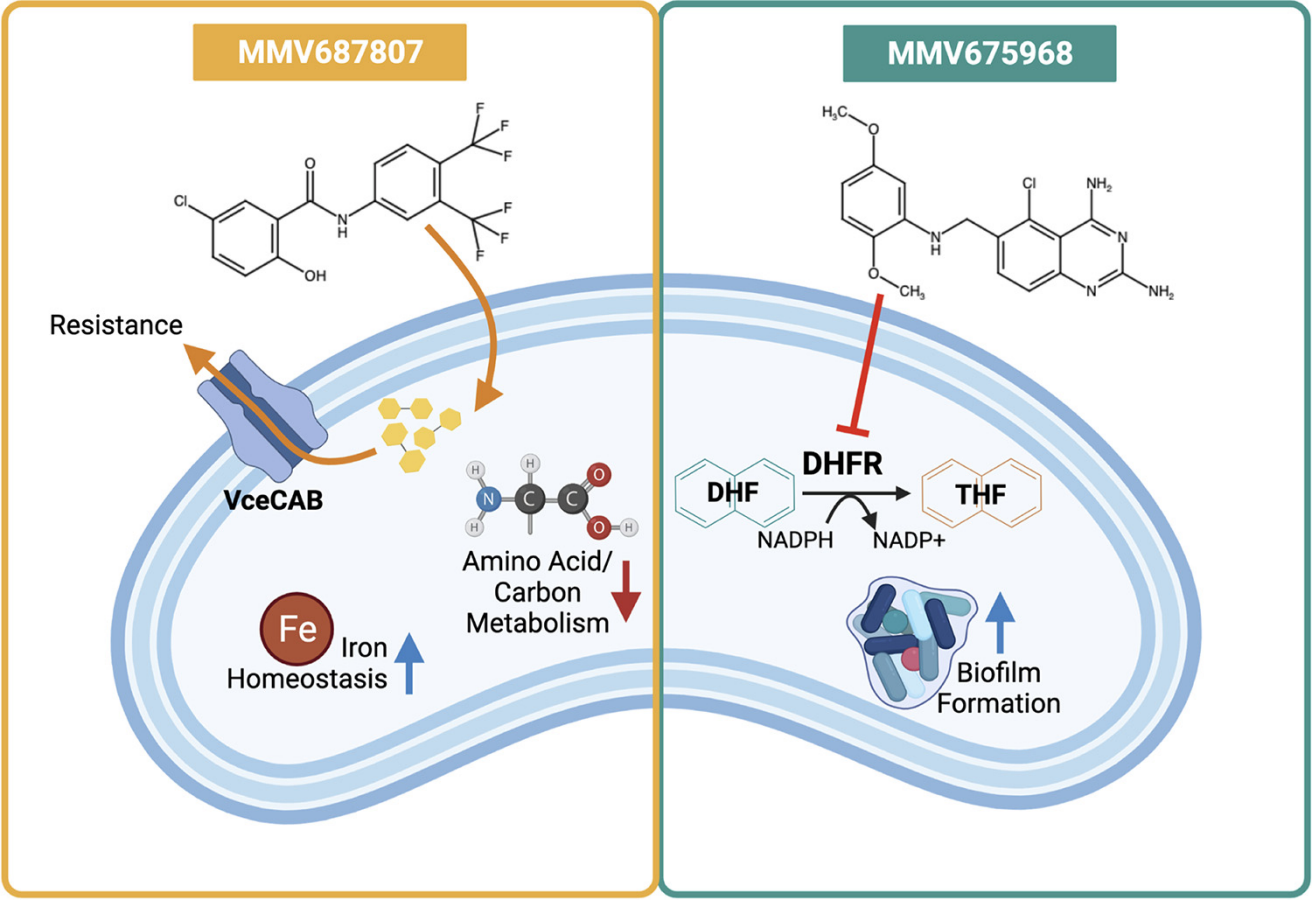
A summary model of each compound’s effect on V. cholerae. MMV687807 causes upregulation of genes involved in iron homeostasis whereas metabolism of amino acids and carbon is downregulated. Efflux pump VceCAB expels the compound leading to resistance. MMV675968 causes genes involved in biofilm formation to be upregulated. The compound inhibits the activity of DHFR, enzyme involved in nucleic acid synthesis, causing growth inhibition of the cells.

In summary, we have identified two compounds, MMV687807 and MMV675968, that effectively inhibit the growth of V. cholerae (Fig. 5). Future research will be focused on determining the target of MMV687807, structurally characterizing the interaction between MMV675968 and DHFR, and their efficacy through *in vivo* models. Collectively, these findings will facilitate not only the treatment of cholera but also the discovery of novel compounds against broad multi-drug resistant infections.

## MATERIALS AND METHODS

### Growth conditions.

Unless otherwise stated, V. cholerae El Tor strain C6706 was aerobically grown at 37°C in LB (wt/vol 1% tryptone, 0.5% yeast extract, 0.5% NaCl) shaking at 225 rpm or on LB-agar plates with indicated concentrations of compound.

### Pathogen Box screening against V. cholerae.

The Pathogen Box molecules were diluted to a concentration of 1 mM in DMSO and used at 10 μM in LB for screening. Then, 200 μL of V. cholerae cell culture with OD_600_ ∼1 was spread on 15-cm diameter LB-agar plates as bacterial lawns and left to dry. Also, 5 μL of each Pathogen Box molecule at 10 μM concentration was spotted on the plate using a replicator and the plate was incubated at 37°C overnight. Six Pathogen Box molecules out of 400 tested molecules resulted in a zone of clearing of V. cholerae after overnight growth on solid LB-agar. These six molecules (Table 1) were re-tested for inhibition of V. cholerae growth on LB-agar plates.

### Growth curve with Pathogen Box compounds.

C6706 was grown overnight and diluted to OD_600_ ∼0.05 in 200 μL of fresh LB containing indicated concentrations of each compound and DMSO (0.2%) in 96-well plate. The growth was observed every 30 min for 8 h at 37°C by measuring OD_600_. For strains containing plasmid, 0.2% arabinose was used for expression and 0.4% glucose for repression.

### CFU/mL counting with Pathogen Box compounds.

V. cholerae was grown to mid-logarithmic stage (OD_600_ of 0.5) and then varying concentrations of a Pathogen Box molecule or equivalent percentage of DMSO control (0.05% maximum) were added. The growth was monitored over time by measuring the OD_600_. At each time point, a 10-fold serial dilution of cells was plated on LB-agar and grown overnight at 37°C. Colonies were counted to quantify CFU per mL (CFU/mL).

### CFU/mL counting with stationary C6706 and Pathogen Box compounds.

C6706 was grown overnight for 18 h before being treated with indicated concentrations of each compound. At each time point, 5 μL of 10-fold serial dilutions of C6706 were plated on LB-agar and grown overnight at 37°C. Colonies were counted to quantify CFU per milliliter (CFU/mL).

### MIC assay.

The MIC was determined following the Clinical and Laboratory Standards Institute microdilution assay using the cation-adjusted Mueller-Hinton broth (CAMHB) (22). V. cholerae was grown on solid LB-agar at 37°C overnight. The colonies were resuspended in CAMHB to a 0.5 McFarland standard. The 100 μL of adjusted culture was further diluted with serially diluted concentrations of MMV675968 in CAMHB in a round bottom 96-well plate to a final concentration of ∼5 × 10^5^ CFU/ml. The plate was grown at 37°C for 18 h. The lowest concentration with no visible growth was recorded and averaged over three biological replicates to identify the MIC.

### RNA purification from V. cholerae C6706.

For RNA transcriptome analysis, C6706 was grown to an OD_600_ of 0.4. Cells were then incubated with sub-inhibitory concentrations of MMV687807 (2.5 μM), MMV675968 (10 nM), or DMSO control (0.2%) in triplicate. After 30 min of growth with each concentration of compound or DMSO, 700 μL of cells were harvested for RNA extraction. For RNA extraction, 700 μL of cells were incubated with 100 μL of 8x lysis buffer (0.8% SDS and 16 mM EDTA) and an equal volume of acidic phenol and incubated at 65°C for 5 min, followed by incubation on ice for 10 min. After phase separation by centrifugation for 2 min at 13,000 × *g*, the aqueous phase was isolated and combined with an equal volume of anhydrous ethanol. RNA was purified using the Direct-zol RNA miniprep kit (Zymo Research) with an in-column DNase I digest to remove contaminating DNA. After quantification of RNA, the rRNA was removed with the RiboZero kit from Illumina.

### RNA-seq transcriptome analysis.

The extracted RNA samples were sequenced at McGill University and Génome Québec Innovation Centre using the HiSeq4000 Illumina sequencer. The resulting reads were 50-nucleotide single-end sequences. Using FastQC (43), each of the raw reads were checked for their per base sequence quality and overrepresented sequences for any adaptor sequence recognition. Following parameters were used with Trimmomatic on Galaxy platform to improve the quality of the sequences including removing any of the recognized adapters: (i) initial ILLUMINACLIP with customized adapter sequences, (ii) filtering out poor quality sequences using pre-set parameters of sliding window, leading, and trailing functions, and (iii) heading cropping sequences improving per base sequence content.

The quality checked sequences for each sample were mapped against the genomic sequence of V. cholerae O1 biovar El Tor str. N16961 which were calculated for expression levels and normalized using the Geneious software version 10.1.3. DESeq2 was used to calculate the differential expression between DMSO control and each of the compound-treated samples. For MMV687807-treated reads, genes with differential expression log_2_ value greater than 2 were considered upregulated whereas genes with differential expression log_2_ value less than −2 were considered downregulated. On the other hand, genes from MMV675968-treated samples had log_2_ value greater than 1 and less than −1 as its cutoffs. Both sets of genes were considered statistically significant if the adjusted *P value* was <0.05. The biological pathways of upregulated or downregulated genes were identified using KEGG pathway mapper and UniProtKB.

### Light microscopy acquisition.

C6706 was grown to an OD_600_ of 0.6 before the addition of each indicated compound. At each time point, cells were concentrated to an OD_600_ ∼5, resuspended in 0.5X PBS, and spotted on 1% agarose-0.5X PBS pads. All the phase-contrast images were obtained using a Nikon Ti-E inverted microscope with a 1 Perfect Focus System 2 (PFS) and a CFI Plan Apochromat Lambda 100X oil objective lens. Three separate fields for each treatment in individual replicate were obtained. Fiji software was used for image manipulation to have equal contrast between the compared images. Each panel is a representative image from three biological replicates.

### Biofilm assay.

C6706 was grown overnight and subcultured to reach OD_600_ of 0.6. Subculture was mixed 1:1 in a 96-well plate with LB containing indicated concentration of MMV675968. Cells were grown at room temperature for 20 h without shaking. Floating cells and LB were removed, and the biofilm was washed with deionized water twice then airdried. Biofilm was stained with 200 μL of 0.1% crystal violet for 10 min. Excess crystal violet was removed, and the wells were washed twice with deionized water then airdried. Crystal violet was resuspended with DMSO and measured at OD_595_.

### Spontaneous repressor mutants.

Overnight culture of wildtype C6706 was adjusted to OD_600_ = 1. 200 μL of the adjusted culture was spread as bacterial lawn on LB-agar plates containing 3 μM or 5 μM MMV687807 or 25 nM or 50 nM MMV675968. Plates were then incubated at 37°C overnight. Resistance of colonies that grew was further confirmed by growing collected colonies on new LB-agar plates with appropriate concentrations of each compound.

### Whole genome sequencing and analysis.

Total DNA was extracted with DNeasy blood and tissue kit (Qiagen) from an overnight culture grown from a single colony of wildtype or resistant mutant strains. DNA was quantified using Qubit 4.0. NEBNext Ultra II DNA Library Prep Kit for Illumina was used for library preparation. High through-put genome sequencing was carried out at the McGill University and Génome Québec Innovation Centre (*n* = 6) on Illumina HiSeq4000 platforms, generating 100 bp single-end reads, resulting in a mean of 1,625-fold coverage. Short reads of wildtype C6706 were assembled with Unicycler version 0.4.4 and annotated with Prokka v1.12 with default parameters. A reference-based alignment for each of the resistant mutant strain was generated by mapping Illumina-generated short reads of resistant mutants to *de novo* assembly of wildtype C6706 from above using Snippy v3.2-dev. The predicted mutations were further confirmed to be causing resistance by growing transposon mutants of the genes in indicated concentrations of each compound following the growth curve assay.

### Diagrams.

Schematic of the screening (Fig. 1A) and the summarizing cell model (Fig. 5) was created with BioRender.com.

### Data availability.

The raw sequence reads of RNA-seq have been deposited to NCBI’s Gene Expression Omnibus (GEO) under the accession number GSE185596 (https://www.ncbi.nlm.nih.gov/geo/query/acc.cgi?acc=GSE185596). The raw sequence reads of whole-genome sequencing have been deposited to Sequence Read Archive (SRA) with the accession number PRJNA771235.
