# Supplementary material for: Identification of Small Molecule Inhibitors of the Pathogen Box against Vibrio cholerae
Source: Microbiol Spectr. 2021 Dec 22;9(3):e00739-21. doi: 10.1128/Spectrum.00739-21 (PMC8694189; doi:10.1128/Spectrum.00739-21)
Supplement: SUPPLEMENTAL FILE 1 — Supplemental material. Download SPECTRUM00739-21_Supp_1_seq11.pdf, PDF file, 0.3 MB [file spectrum00739-21_supp_1_seq11.pdf]

Supplementary Data

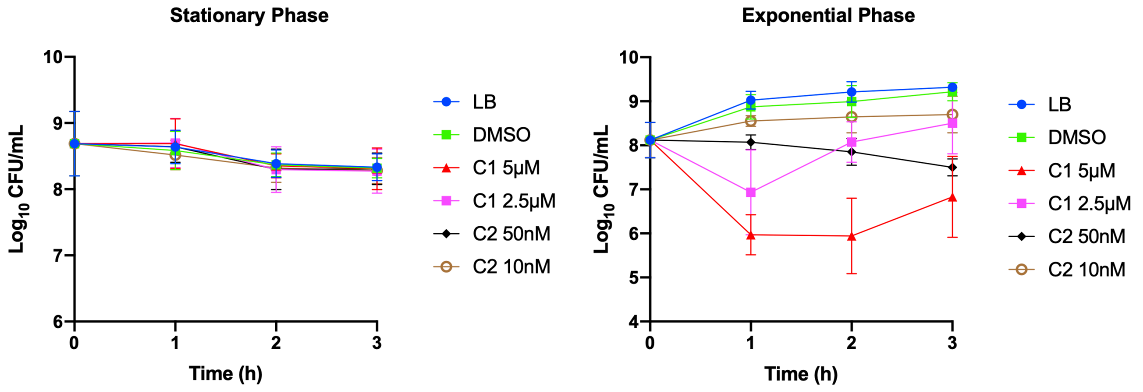

Supplementary Figure 1. Both MMV687807 and MMV675968 targets actively growing cells. OD_{600} growth curve measurements of C6706 cells killing with varying concentrations of MMV687807 (C1) or MMV675968 (C2) at stationary or exponential phase. Cells were grown for ~18h or to an OD_{600} of 0.6 before the addition of each compound or DMSO. 10-fold serial dilution of cells was plated on LB-agar for counting colonies at each time point. Error bars indicate mean ± standard deviation of four biological replicates.

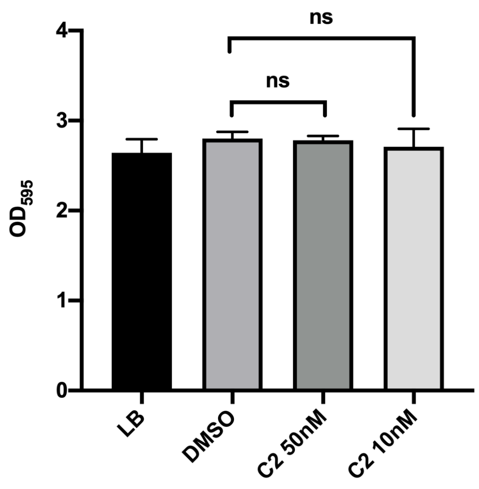

Supplementary Figure 2. Biofilm formation is not affected by exposure to MMV675968. C6706 were grown to OD_{600} ~0.6 and grown in fresh LB for 20h at room temperature. Biofilm was quantified by measuring OD_{595} of crystal violet-stained biofilm resuspended in DMSO. One-way

ANOVA with Sidak's multiple comparison was used to compare treatments, ns, not significant. Error bars indicate mean ± standard deviation of three biological replicates.

Supplementary Table 1. List of genes that exhibited differential expression with adjusted p-value less than 0.05 for each compound.

Supplementary Table 2. A list of genes detected in both RNA-seq analyses of cell treated with either MMV687807 or MMV675968.

Supplementary Table 3. Whole-genome sequence analysis of spontaneous repressor mutants and the list of genes mutated.
